# Biomaterial-based strategies for osteoporosis treatment and bone regeneration: advances and translational challenges

**DOI:** 10.3389/fbioe.2026.1762509

**Published:** 2026-07-20

**Authors:** Xiaoqin Qiu, Ya Ren

**Affiliations:** 1 Department of Oncology, Cancer Prevention and Treatment Institute of Chengdu, Chengdu Fifth People’s Hospital (The Second Clinical Medical College, Affiliated Fifth People’s Hospital of Chengdu University of Traditional Chinese Medicine), Chengdu, China; 2 Department of Biotherapy Cancer Center and State Key Laboratory of Biotherapy, West China Hospital, Sichuan University, Chengdu, China

**Keywords:** biomaterials, bone regeneration, injectable hydrogel, osteoporosis, osteoporotic bone defect, scaffold, targeted drug delivery

## Abstract

Osteoporosis is a systemic skeletal disorder characterized by reduced bone mass, deterioration of bone microarchitecture, and increased susceptibility to fragility fractures. Although conventional antiresorptive and anabolic drugs effectively reduce fracture risk in many patients, their clinical utility is restricted by poor tissue specificity, systemic adverse effects, adherence problems, discontinuation-related risks, and their limited capacity to regenerate osteoporotic bone defects after trauma or surgery. Biomaterial-based strategies provide complementary opportunities by combining local structural support, controlled therapeutic delivery, and microenvironmental regulation. In this review, we discuss biomaterial design from an osteoporosis-specific perspective, emphasizing how disease-associated abnormalities—impaired osteoblast function, excessive osteoclast activity, reduced angiogenesis, inflammatory dysregulation, compromised extracellular matrix quality, and weakened mechanical integrity—can be addressed by scaffolds, targeted drug delivery systems, and biologically derived platforms. Ceramic, polymeric, and composite scaffolds are compared with respect to osteoconduction, ion-mediated signaling, mechanical support, and manufacturability. Bone-targeted nanoparticles, injectable hydrogels, and stimuli-responsive carriers are evaluated as strategies for the localized delivery of antiresorptive agents, anabolic molecules, nucleic acids, and osteogenic cues. We further summarize platelet-rich plasma/platelet-rich fibrin, growth factor-loaded matrices, mesenchymal stem cell–laden scaffolds, extracellular vesicle–functionalized systems, and gene-activated matrices as emerging biological or cell-free regenerative platforms. Finally, key translational barriers, including long-term safety, reproducible manufacturing, standardized osteoporotic models, and regulatory pathways for combination products, are discussed. Overall, biomaterials should not be viewed as replacements for established pharmacotherapy but as disease-tailored local interventions that may improve osteoporotic fracture repair and bone regeneration when integrated with rational clinical management.

## 1 Introduction

Osteoporosis (OP) is a highly prevalent skeletal disease characterized by low bone mass, deterioration of trabecular and cortical microarchitecture, and increased fracture risk (Compston et al., 2019; Rachner et al., 2011). It affects hundreds of millions of people worldwide and represents a major cause of disability, mortality, and healthcare expenditure in aging societies (Compston et al., 2019; Rachner et al., 2011; LeBoff et al., 2022). Osteoporotic fractures are not only a consequence of low bone strength but also a clinical context in which bone repair is frequently compromised. Delayed union, implant loosening, vertebral collapse, and poor integration of grafts or substitutes remain important challenges in osteoporotic patients. Current pharmacological management primarily relies on antiresorptive agents, including bisphosphonates and denosumab, and anabolic agents, including teriparatide, abaloparatide, and romosozumab (Eastell et al., 2019; Shoback et al., 2020; Qaseem et al., 2023). These drugs are indispensable for systemic fracture risk reduction. However, systemic therapy does not directly reconstruct large bone defects, fill irregular fracture voids, or provide mechanical support at compromised sites. Long-term treatment is also constrained by adherence, rare but serious adverse events, duration restrictions, and discontinuation-related risks (Eastell et al., 2019; Shoback et al., 2020; Qaseem et al., 2023; Shane et al., 2014; Cummings et al., 2009; Saag et al., 2017). Therefore, local therapeutic strategies that can complement systemic therapy are increasingly needed.

**FIGURE 1:**
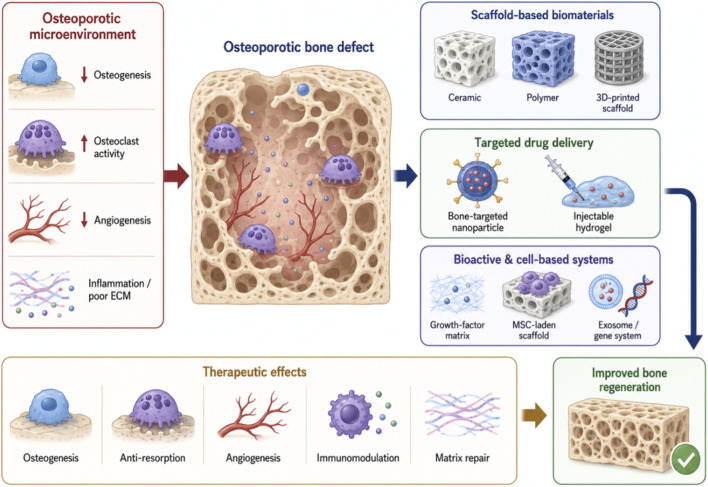
Schematic overview of biomaterial-based strategies for osteoporosis treatment and osteoporotic bone regeneration. Conventional pharmacological therapies reduce fracture risk but have limitations in tissue specificity, local regeneration, and long-term management. Biomaterials may address these limitations through scaffold-based support, localized drug delivery, biologically derived components, and multifunctional platforms.

Biomaterials provide such an opportunity because they can be designed to serve as three-dimensional scaffolds, local drug depots, biological signal reservoirs, or multifunctional platforms. In osteoporotic bone, the material design goal is not simply to induce bone formation in a normal defect; rather, the material must operate in a microenvironment characterized by impaired osteoblast activity, excessive osteoclast-mediated resorption, insufficient vascularization, chronic low-grade inflammation, defective extracellular matrix (ECM) formation, and poor mechanical integrity. A disease-specific framework is therefore essential for evaluating biomaterial strategies in osteoporosis.An overview of the osteoporosis-specific pathological barriers and corresponding biomaterial strategies is provided in Figure 1.

This review emphasizes osteoporosis-specific design logic, representative material systems, and translational challenges. We distinguish between two related but different clinical concepts: systemic osteoporosis treatment, which aims to reduce future fracture risk, and osteoporotic bone defect or fracture repair, which requires local tissue regeneration and mechanical stabilization. Biomaterial-based approaches are most mature in the latter context and may become clinically useful as adjunctive strategies integrated with pharmacological management, surgical fixation, and rehabilitation.

## 2 Literature search strategy

This article was prepared as a narrative review rather than a formal systematic review. To improve transparency and reproducibility, relevant literature was identified from PubMed, Web of Science, and Scopus using combinations of the following terms: osteoporosis, osteoporotic bone defect, fragility fracture, biomaterial, scaffold, bone tissue engineering, bioceramic, bioactive glass, hydroxyapatite (HA), β-tricalcium phosphate (β-TCP), polymer scaffold, composite scaffold, three-dimensional (3D) printing, injectable hydrogel, bone-targeted nanoparticle, bisphosphonate delivery, strontium, magnesium, immunomodulatory biomaterial, platelet-rich plasma, extracellular vesicle (EV), exosome, mesenchymal stem cell (MSC), gene-activated matrix, and controlled drug delivery.

The search focused on English-language original research articles, reviews, guidelines, and translational studies published mainly between 2000 and 2026, with priority given to recent studies and reviews from the past decade. Clinical guidelines and landmark mechanistic studies were included when they provided essential background on osteoporosis therapy, osteoclast biology, bone remodeling, or regulatory considerations. Both *in vitro* studies and preclinical animal studies were considered when they provided mechanistic or functional evidence relevant to biomaterial design. Clinical studies were included when available, but the current biomaterial literature remains dominated by preclinical work.

Studies were selected based on relevance to osteoporosis-specific pathology, biomaterial composition, therapeutic mechanism, experimental model, reported biological outcomes, and translational significance. Because this is a narrative review, the selection was not intended to meet PRISMA standards; instead, representative examples were chosen to illustrate major design principles and emerging trends.

## 3 Pathophysiological rationale for biomaterial design in osteoporosis

Osteoporosis results from an imbalance between bone resorption and bone formation. Under physiological conditions, osteoclast-mediated matrix resorption is coupled with osteoblast-mediated matrix deposition and mineralization. Aging, estrogen deficiency, glucocorticoid exposure, metabolic disease, and chronic inflammation can disrupt this coupling and shift remodeling toward net bone loss (Teitelbaum, 2000; Boyle et al., 2003; Manolagas, 2000; Khosla and Monroe, 2018). Biomaterial design for osteoporosis should therefore consider both cellular dysfunction and the structural fragility of the local bone environment.

At the cellular level, osteoporotic bone typically exhibits reduced osteoblast differentiation and survival, increased osteoclast formation and activity, and altered osteocyte signaling. The RANK/RANKL/OPG axis, Wnt/β-catenin signaling, sclerostin, inflammatory cytokines, and oxidative stress all contribute to impaired remodeling (Teitelbaum, 2000; Boyle et al., 2003; Manolagas, 2000; Khosla and Monroe, 2018; Black and Rosen, 2016). Materials that release osteogenic ions, present osteoinductive surfaces, or deliver anabolic factors may help rescue defective osteogenesis. Conversely, local delivery of antiresorptive agents or ions that suppress osteoclast activity may reduce excessive matrix degradation.

**FIGURE 2:**
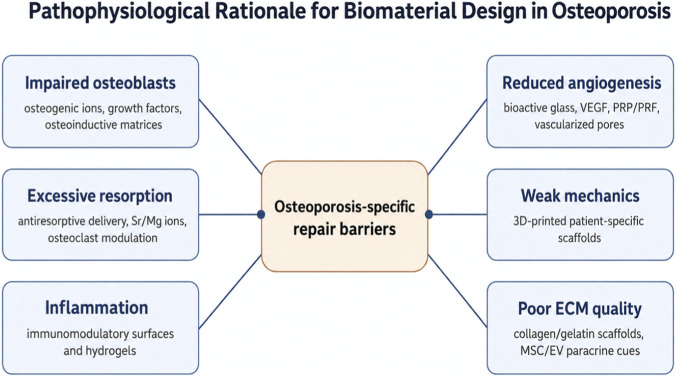
Osteoporosis-specific pathological barriers and corresponding biomaterial design strategies. Osteoporotic bone repair is constrained by impaired osteoblast function, excessive osteoclast activity, reduced angiogenesis, inflammatory dysregulation, poor ECM quality, and insufficient mechanical integrity. Biomaterial strategies can be designed to address these barriers through osteogenic ion release, antiresorptive delivery, immunomodulatory cues, vascularized architecture, ECM-mimetic surfaces, and patient-specific mechanical support.

At the tissue level, osteoporotic bone has diminished vascular support, compromised collagen matrix quality, increased porosity, and reduced mechanical strength. These features are directly relevant to scaffold design. An ideal material should provide mechanical support without stress shielding, guide vascular invasion, promote organized ECM deposition, and allow gradual load transfer to newly formed bone. The same biomaterial may also modulate inflammation because immune dysregulation contributes to bone loss and can impair the transition from early inflammation to constructive repair. This immunomodulatory rationale is supported by recent biomaterial studies (Xie et al., 2020; Zhao et al., 2022). The major osteoporosis-specific pathological barriers and corresponding biomaterial strategies are summarized in Figure 2. Accordingly, osteoporosis-oriented biomaterial design should link a disease abnormality with a specific material function rather than simply classifying materials by composition. Table 1 summarizes this design logic.

**TABLE 1:** Osteoporosis-related pathological features and corresponding biomaterial design strategies.

Osteoporosis-related abnormality	Biomaterial strategy	Design mechanism	Representative example
Impaired osteoblast function	Osteoinductive scaffolds and anabolic delivery	Enhance osteoblast differentiation, matrix deposition, and mineralization	Sr-, Mg-, or Si-doped bioceramics; BMP-2; and PTH fragment or simvastatin delivery systems
Excessive osteoclast-mediated resorption	Local antiresorptive delivery and ion-mediated modulation	Suppress osteoclastogenesis or reduce resorptive activity while limiting systemic exposure	Bisphosphonate-loaded nanoparticles or hydrogels, and Sr-containing calcium phosphate or bioactive glass
Reduced angiogenesis	Pro-angiogenic scaffolds and vascularized pore architecture	Improve blood vessel invasion, nutrient exchange, and osteogenesis-angiogenesis coupling	Bioactive glass, VEGF delivery, PRP/PRF, and interconnected 3D-printed channels
Inflammatory dysregulation	Immunomodulatory biomaterials	Promote transition from destructive inflammation to pro-regenerative immune responses	Anti-inflammatory hydrogels, macrophage-modulating surfaces, and ROS-responsive systems
Compromised ECM quality	ECM-mimetic materials and cell/EV paracrine cues	Promote collagen-rich matrix formation and subsequent mineralization	Collagen, gelatin, and chitosan scaffolds, MSC-laden scaffolds, and EV- or exosome-functionalized matrices
Weak mechanical integrity	Patient-specific and mechanically tunable scaffolds	Provide temporary support, controlled porosity, and load sharing during repair	3D-printed ceramic/polymer composites, hierarchical porous scaffolds, and shape-adaptive or injectable constructs

## 4 Limitations of conventional osteoporosis therapies

Conventional osteoporosis therapy remains the standard of care for reducing systemic fracture risk. Bisphosphonates inhibit osteoclast-mediated bone resorption and are widely used as first-line agents in patients at high fracture risk (Eastell et al., 2019; Shoback et al., 2020; Qaseem et al., 2023). Denosumab, a monoclonal antibody against RANKL, suppresses osteoclast formation and activity but requires scheduled administration because bone turnover may rebound after discontinuation (Cummings et al., 2009). Anabolic therapies, including teriparatide and abaloparatide, stimulate bone formation, whereas romosozumab inhibits sclerostin and exerts both anabolic and antiresorptive effects (Eastell et al., 2019; Shoback et al., 2020; Saag et al., 2017). Selective estrogen receptor modulators such as raloxifene and hormone-related treatments are useful in selected populations but have specific risk profiles (Eastell et al., 2019; Shoback et al., 2020; Qaseem et al., 2023).

Despite clinical efficacy, these drugs do not directly solve local reconstructive problems. A patient with an osteoporotic fracture or bone defect may need space filling, mechanical support, vascularized tissue formation, and local biological stimulation in addition to systemic risk reduction. Systemic drugs also achieve limited local concentrations in bone defects and may expose non-skeletal tissues to unwanted effects. Rare complications, including atypical femoral fractures and osteonecrosis of the jaw after long-term antiresorptive therapy, are important concerns in risk–benefit assessment (Qaseem et al., 2023; Shane et al., 2014). Romosozumab is generally restricted to selected patients at very high-risk and has cardiovascular cautions (Shoback et al., 2020; Saag et al., 2017). Biomaterials therefore should be framed as complementary local interventions rather than replacements for pharmacological treatment. Their potential value lies in concentrating therapeutic cues at the repair site, reducing systemic exposure, providing structural support, and integrating multiple biological functions into one platform. Table 2 summarizes major drug classes and possible biomaterial-based solutions.

**TABLE 2:** Conventional osteoporosis therapies and potential biomaterial-based solutions.

Drug class	Representative agent	Mechanism of action	Major clinical limitation	Potential biomaterial-based solution
Bisphosphonates	Alendronate, risedronate, ibandronate, and zoledronate	Bind bone mineral and inhibit osteoclast-mediated resorption	Gastrointestinal intolerance for oral agents; renal considerations; rare osteonecrosis of the jaw and atypical femoral fractures; limited local regenerative effect	Bone-targeted nanoparticles or injectable depots for localized and sustained release and incorporation into scaffolds for osteoporotic defects
RANKL inhibitor	Denosumab	Blocks RANKL and suppresses osteoclast formation and function	Scheduled administration required; rebound bone turnover and vertebral fracture risk after discontinuation; hypocalcemia risk	Local antiresorptive release combined with osteogenic scaffolds; strategies that avoid abrupt systemic discontinuation effects require caution
PTH analogs	Teriparatide and abaloparatide	Intermittent PTH receptor stimulation enhances bone formation	Daily injection; duration limits; cost; systemic exposure; not a structural repair material	PTH fragment-loaded hydrogels or scaffolds for local anabolic stimulation in bone defects
Sclerostin inhibitor	Romosozumab	Promotes bone formation and reduces resorption through sclerostin inhibition	Generally limited to 1 year; cardiovascular caution in high-risk patients	Local Wnt-modulating strategies remain experimental; biomaterials may provide local osteoinductive cues without systemic exposure
SERMs and hormone-related therapies	Raloxifene and estrogen-related therapies	Modulate estrogen receptor signaling and reduce bone resorption in selected patients	Thromboembolic and patient-selection concerns; limited defect repair capacity	Adjunctive local biomaterials may be needed for fracture repair or defect reconstruction
Calcitonin and supportive agents	Calcitonin and calcium/vitamin D supplementation	Reduce resorption modestly or support mineral metabolism	Limited antifracture efficacy for some agents; supportive rather than regenerative	Scaffolds and delivery systems can provide local structural and biological functions not achieved by supplementation alone

## 5 Scaffold-based biomaterials for osteoporotic bone regeneration

**FIGURE 3:**
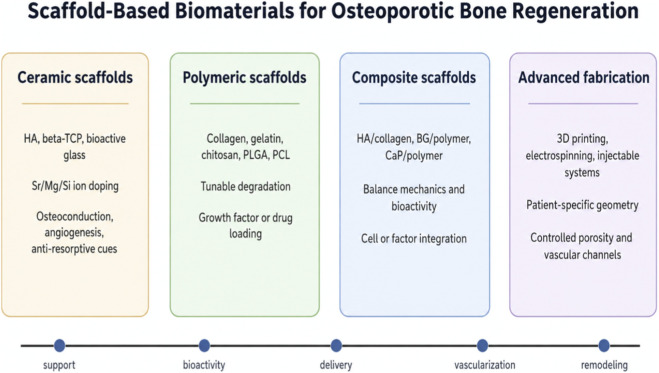
Revised classification of scaffold-based biomaterials for osteoporotic bone regeneration. Ceramic scaffolds mainly provide osteoconduction and ion-mediated signaling; polymeric scaffolds offer tunable degradation and drug-loading capability; composite scaffolds integrate the mechanical and biological advantages of both inorganic and organic components; and advanced fabrication approaches such as 3D printing and electrospinning enable patient-specific architecture, controlled porosity, and vascular channels. This classification separates hybrid composite systems from purely ceramic materials to avoid conceptual overlap.

Scaffold-based biomaterials aim to provide a temporary framework for bone ingrowth, ECM deposition, and remodeling. In osteoporosis, scaffold design must address poor bone quality and reduced repair capacity. Composition, architecture, degradation, surface chemistry, mechanical properties, and biological functionalization are all critical. The following subsections classify these materials to avoid overlap among ceramic, polymeric, and composite systems. The main scaffold categories and advanced fabrication approaches are summarized in Figure 3.

### 5.1 Ceramic-based scaffolds

Calcium phosphate ceramics, including HA and β-TCP, and bioactive glasses are widely used for bone regeneration because their mineral composition resembles that of native bone (Dorozhkin, 2009; Hench, 1998; Hoppe et al., 2011). These materials support osteoconduction, mineral deposition, and direct bonding to bone. However, a purely osteoconductive material may be insufficient in an osteoporotic environment, where endogenous osteogenic activity is reduced and resorption is excessive.

Ion-doped ceramics are therefore particularly relevant to osteoporosis. Strontium can simultaneously promote osteoblast differentiation and suppress osteoclast activity, making Sr-incorporated ceramics attractive for osteoporotic defects (Wu et al., 2022; Chen et al., 2021; Alves Côrtes et al., 2024). For example, Sr-incorporated amino-functionalized mesoporous bioactive glass scaffolds improved osteogenesis and angiogenesis *in vitro* and enhanced bone regeneration and vessel formation in an osteoporotic rat model (Wu et al., 2022). Magnesium- and silicon-containing ceramics may also promote osteogenesis and vascularization, although their degradation rates, local ion concentrations, and long-term safety must be carefully optimized. Bioactive glass-based materials provide additional advantages because their dissolution products can stimulate osteogenic and angiogenic responses. Nonetheless, brittleness and limited toughness remain important limitations for load-bearing defects. In osteoporotic patients, where fixation stability is already compromised, ceramic scaffold mechanics must be balanced with degradability and surgical handling.

### 5.2 Polymeric scaffolds

Natural polymers such as collagen, gelatin, chitosan, alginate, and hyaluronic acid mimic aspects of the ECM and can provide cell-adhesive or enzymatically degradable environments. Synthetic polymers such as poly (lactic-co-glycolic acid), polycaprolactone, and polyurethane offer tunable mechanical properties, processability, and degradation profiles (Rezwan et al., 2006; O'Brien, 2011; Bose et al., 2012; Li and Mooney, 2016). In osteoporotic bone repair, polymeric scaffolds can be used as matrices for osteogenic cells, growth factors, antiresorptive drugs, or nanoparticles. Hydrogels are particularly useful for minimally invasive delivery because they can conform to irregular defects and provide hydrated ECM-like microenvironments (Li and Mooney, 2016; Tang et al., 2025). However, many hydrogels are mechanically weak and require reinforcement when used in load-bearing skeletal sites. Their degradation and release kinetics must also match the temporal sequence of osteoporotic repair, where early inflammation, vascularization, matrix deposition, and mineralization may be delayed.

### 5.3 Composite scaffolds

Composite scaffolds combine inorganic and organic phases to overcome the limitations of single-component materials. Examples include HA/collagen composites, bioactive glass/polymer hybrids, calcium phosphate/polymer scaffolds, and ceramic–polymer matrices loaded with cells or bioactive molecules (Gentleman et al., 2010; Rezwan et al., 2006; O'Brien, 2011; Bose et al., 2012). The inorganic phase improves osteoconductivity and stiffness, whereas the polymeric phase improves toughness, processability, and drug-loading capacity. For osteoporosis-related defects, composite design is especially attractive because mechanical fragility and biological insufficiency coexist. A composite scaffold can be tuned to provide initial structural stability while releasing ions, drugs, or growth factors. Composite systems also allow the incorporation of stem cells, extracellular vesicles, or gene vectors, thereby integrating scaffold, delivery, and biological functions. The major challenges are reproducible fabrication, sterilization, quality control, and prediction of degradation behavior in elderly or metabolically compromised patients.

### 5.4 Advanced fabrication: 3D printing and electrospinning

Additive manufacturing enables patient-specific scaffolds with defined external geometry, internal porosity, pore interconnectivity, and mechanical anisotropy (Murphy and Atala, 2014; Zadpoor, 2015; Turnbull et al., 2018; Qi et al., 2025).Recent reviews further highlight advances in 3D-printed and responsive scaffolds for bone repair (Wang et al., 2024; Bai et al., 2025). These features are clinically relevant because osteoporotic defects often have irregular shapes and require balanced load sharing. 3D-printed scaffolds can also incorporate gradients in pore size, stiffness, or bioactive components to promote vascularized bone formation. In the future, computational modeling and artificial intelligence may help match scaffold architecture to patient-specific defect geometry and local mechanical requirements. Electrospinning produces fibrous matrices that resemble collagen networks and can be used as membranes, coatings, or hybrid scaffold components. Nanofibrous surfaces may enhance cell adhesion and ECM deposition, but electrospun mats alone usually lack sufficient three-dimensional thickness and mechanical strength for large defects. Combining electrospun layers with 3D-printed frameworks may provide both microstructural biomimicry and macroscale mechanical support.

## 6 Biomaterial-based targeted drug delivery systems

**FIGURE 4:**
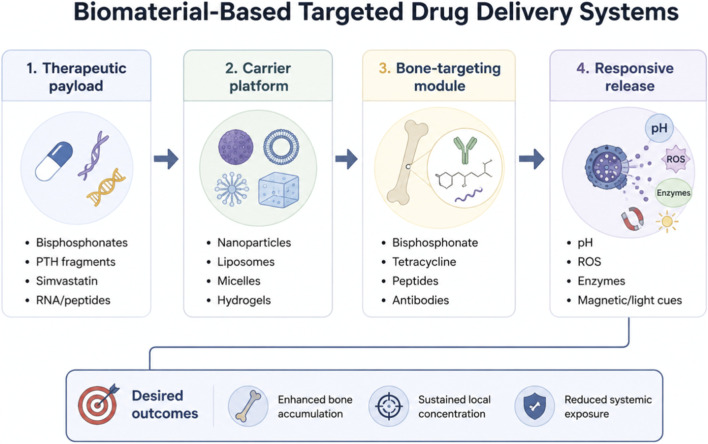
Key components of biomaterial-based targeted drug delivery systems for osteoporosis-related applications. A delivery system can be constructed by combining therapeutic payloads, carrier platforms, bone-targeting ligands, and responsive release mechanisms. Desired outcomes include enhanced bone accumulation, sustained local concentration, reduced systemic exposure, and improved repair of osteoporotic defects. Representative payloads include bisphosphonates, PTH fragments, simvastatin, nucleic acids, and osteogenic peptides.

Targeted drug delivery systems (DDS) are designed to increase therapeutic concentration at bone sites while reducing systemic exposure. In osteoporosis, DDS can deliver antiresorptive drugs, anabolic molecules, osteogenic small molecules, nucleic acids, or immunomodulatory agents. Bone-targeting strategies often exploit ligands with high affinity for hydroxyapatite, such as bisphosphonates, tetracycline derivatives, and acidic peptides (Wen et al., 2024; Wu et al., 2024). Carrier platforms include polymeric nanoparticles, liposomes, micelles, dendrimers, inorganic nanoparticles, and injectable hydrogels. The key components of biomaterial-based targeted drug delivery systems are illustrated in Figure 4, and representative targeted delivery approaches are summarized in Table 3.

**TABLE 3:** Representative targeted delivery approaches for osteoporosis-related bone regeneration.

Delivery system	Therapeutic agent or cargo	Targeting/release mechanism	Model or application context	Main reported outcome
Bone-targeted nanoparticles	Bisphosphonates, anabolic drugs, and nucleic acids	Hydroxyapatite-binding ligands such as bisphosphonates, tetracycline derivatives, or acidic peptides	Systemic or local osteoporosis-related therapy in preclinical studies	Enhanced bone accumulation and potential reduction of systemic exposure
Alendronate-functionalized double-network hydrogel	Alendronate incorporated into GelMA/oxidized alginate network	Schiff-base-related pH-responsive degradation and sustained release	*In vitro* bone regeneration model and scaffold evaluation	Improved biomechanics, biocompatibility, and osteogenic gene expression
Dual-drug biomimetic delivery system	PTH1-34 and simvastatin	Controlled local release from biomimetic carrier	*In situ* osteoporotic bone regeneration	Promoted osteogenic differentiation and bone regeneration
Calcium phosphate or bone cement carriers	Bisphosphonates or osteogenic molecules	Local depot release with osteoconductive mineral phase	Osteoporotic fracture or bone defect filling	Potential reinforcement and local modulation of remodeling
Stimuli-responsive nanoparticles or hydrogels	Small molecules, peptides, RNA, or proteins	pH-, ROS-, enzyme-, magnetic-, or light-responsive release	Emerging preclinical smart DDS	Improved spatiotemporal control but requires safety and regulatory validation

Nanoparticle-based DDS can be engineered for size, surface charge, ligand density, degradation, and release kinetics. Bone-targeted nanoparticles for osteoporosis have been reviewed in detail, with bisphosphonates and related mineral-binding groups commonly used to direct carriers toward bone mineral (Wen et al., 2024; Wu et al., 2024). The therapeutic advantage is not limited to targeting; nanoparticles can also protect unstable cargo, prolong circulation time, co-deliver multiple agents, and respond to local stimuli. Injectable hydrogels are a second major DDS category. They can fill irregular defects, retain cargo locally, and release bioactive molecules over time (Li and Mooney, 2016; Tang et al., 2025). For example, an alendronate-functionalized double-network hydrogel based on GelMA and oxidized alginate was reported to provide pH-responsive degradation, sustained alendronate release, improved mechanical properties, and enhanced osteogenic differentiation (Tang et al., 2022). A personalized dual-drug system delivering PTH1-34 and simvastatin was developed for *in situ* osteoporotic bone regeneration, illustrating the potential of combining anabolic and osteogenic small molecule cues (Xu et al., 2024). Stimuli-responsive DDS further enhance precision by releasing cargo in response to acidic pH, reactive oxygen species, enzymes, magnetic fields, or other cues associated with the bone microenvironment (Sun et al., 2017). In osteoporotic repair, pH-responsive release may be relevant to osteoclast-rich resorption lacunae, whereas ROS-responsive materials may address oxidative stress and inflammation. Nonetheless, most smart DDS remain preclinical, and their safety, biodistribution, degradation products, and manufacturing reproducibility require systematic evaluation.

## 7 Biologically derived and cell-based biomaterials

**FIGURE 5:**
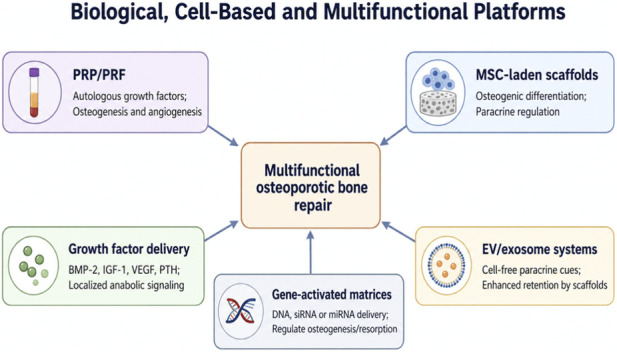
Biologically derived, cell-based, and multifunctional biomaterial platforms for osteoporotic bone regeneration. PRP/PRF provides autologous growth factors; growth factor matrices support localized anabolic signaling; MSC-laden scaffolds provide cells and paracrine effects; EV/exosome systems offer cell-free regulatory cues; and gene-activated matrices may regulate osteogenesis or osteoclastogenesis. These systems are often most effective when integrated with structural scaffolds and controlled delivery platforms.

Biologically derived biomaterials provide endogenous signals that can complement synthetic or mineral scaffolds. Platelet-rich plasma (PRP) and platelet-rich fibrin (PRF) contain platelet-derived growth factor, transforming growth factor-β, vascular endothelial growth factor, and other cytokines that can support osteogenesis and angiogenesis (Marx, 2004; Amiri et al., 2023). Representative biologically derived, cell-based, and multifunctional platforms are summarized in Figure 5. In osteoporotic contexts, PRP/PRF may partially compensate for impaired local healing capacity, particularly when combined with structural scaffolds or graft substitutes. Variability in preparation protocols, platelet concentration, leukocyte content, and release kinetics remains a major challenge.

Growth factor-loaded matrices represent another important strategy. BMP-2, IGF-1, VEGF, PTH fragments, and related bioactive molecules can be locally delivered using collagen sponges, hydrogels, nanoparticles, or composite scaffolds (Li and Mooney, 2016; Chen et al., 2010). Local delivery can reduce systemic exposure and maintain therapeutic concentrations at the defect site. However, growth factor dose, burst release, ectopic tissue formation, cost, and regulatory requirements must be carefully controlled. A major goal is to design delivery systems that reproduce the temporal sequence of bone healing rather than providing a single uncontrolled bolus. Cell-based scaffolds, especially those incorporating MSCs, can enhance bone repair through both osteogenic differentiation and paracrine signaling (Caplan, 1991). In osteoporotic bone, MSC function may be impaired by age and disease, so the source, preconditioning, and microenvironmental support of the cells are important. Scaffold stiffness, ligand presentation, degradation, and oxygen diffusion all influence cell survival and lineage commitment. Although cell-laden scaffolds are mechanistically attractive, clinical translation is limited by cell processing, reproducibility, storage, cost, and regulatory complexity. EV- or exosome-functionalized scaffolds have emerged as cell-free alternatives. EVs can carry proteins, lipids, mRNA, and microRNA that regulate osteogenesis, angiogenesis, and immune responses. Scaffold delivery may improve EV retention and protect them from rapid clearance (Liu et al., 2023; Yu et al., 2024; Yan et al., 2020). This approach is promising for osteoporotic bone regeneration because it could capture MSC paracrine benefits without using living cells. However, EV heterogeneity, potency assays, large-scale production, and storage remain unresolved issues.

## 8 Combination and multifunctional biomaterial platforms

Because osteoporosis involves multiple pathological abnormalities, single-function materials may be insufficient. Multifunctional platforms can combine mechanical support, osteoinduction, antiresorptive activity, angiogenesis, immunomodulation, and controlled degradation. For example, a 3D-printed composite scaffold can provide defect-specific mechanical support while releasing Sr ions, growth factors, or antiresorptive drugs. Similarly, a hydrogel can co-deliver an anabolic factor and a small molecule while conforming to an irregular defect.

Combination design should be mechanistically rational. An osteoporotic defect may require early inflammation resolution, followed by vascular invasion, osteoblast recruitment, collagen matrix formation, and mineralization. Delivering all factors simultaneously may not reproduce this sequence. Sequential or responsive release systems, multilayer scaffolds, and spatially patterned architectures may better match the biology of bone repair. The most promising systems are likely to integrate temporally controlled biological cues with mechanically competent scaffolds. Gene-activated matrices add another layer of multifunctionality by delivering plasmid DNA, siRNA, mRNA, or microRNA to regulate osteogenic or osteoclastogenic pathways (D’Mello et al., 2017). In principle, these systems can upregulate osteogenic transcription factors, suppress inflammatory signaling, or modulate the RANKL/OPG balance. However, delivery efficiency, off-target effects, vector safety, and regulatory complexity must be resolved before clinical translation. Table 4 summarizes representative biomaterial systems by material component, therapeutic cargo, mechanism, model context, and outcome. This type of evidence-oriented comparison may help readers distinguish conceptual categories from experimentally validated strategies.

**TABLE 4:** Representative biomaterial systems, mechanisms, models, and outcomes relevant to osteoporosis-related bone regeneration.

Biomaterial system	Therapeutic component	Mechanistic rationale	Representative model or context	Outcome emphasis
Sr-incorporated mesoporous bioactive glass scaffold	Sr ions	Promotes osteogenesis and angiogenesis while reducing oxidative stress and osteoclast-related imbalance	Osteoporotic rat model and critical-size defect evaluation	Enhanced bone regeneration, vessel formation, and bone quality parameters
Sr- or Mg-containing calcium phosphate/ceramic scaffold	Osteogenic ions	Dual regulation of osteoblast and osteoclast activity; improved mineral integration	Osteoporotic bone defect repair in preclinical models	Improved new bone formation and osseointegration
Ceramic–polymer composite scaffold	HA, β-TCP, or bioactive glass plus polymer matrix	Combines osteoconduction with toughness, processability, and delivery capacity	Bone defect repair, including compromised bone environments	Improved mechanical-biological balance compared with single components
Injectable hydrogel DDS	Alendronate, simvastatin, PTH fragments, or growth factors	Minimally invasive filling and sustained local release	Osteoporotic fracture or irregular bone defect models	Improved osteogenic markers, callus quality, or bone regeneration
PRP/PRF-scaffold composite	Platelet-derived cytokines and fibrin network	Autologous osteogenic and angiogenic signaling	Osteoporotic bone defect or implant integration studies	Accelerated early healing and vascularized repair
MSC-laden scaffold	MSCs and paracrine factors	Direct osteogenic differentiation and secretion of trophic factors	Preclinical osteoporotic bone repair	Enhanced bone formation compared with the scaffold alone
EV/exosome-functionalized scaffold	MSC-derived or other EVs	Cell-free transfer of osteogenic, angiogenic, and immunomodulatory cargo	Critical-size or osteoporotic bone defect models	Improved EV retention, angiogenesis, and bone repair
Gene-activated matrix	DNA, siRNA, miRNA, or mRNA	Local regulation of osteogenesis, angiogenesis, or osteoclastogenesis	Experimental bone tissue engineering platforms	Promising but requires vector safety and potency validation

## 9 Translational challenges and future perspectives

Despite rapid progress, biomaterial-based strategies for osteoporosis remain largely preclinical. The first challenge is long-term safety. Osteoporotic patients are often elderly and may have metabolic disease, renal impairment, cardiovascular risk, or chronic inflammation. Degradation products, local ion concentrations, immune reactions, delayed remodeling, and interactions with systemic osteoporosis drugs must be evaluated in clinically relevant models. Short-term bone formation is not sufficient; long-term integration, mechanical stability, and remodeling quality are equally important (Williams, 2008; Hench and Polak, 2002).

A second challenge is model selection and standardization. Many studies use ovariectomized rodents, which capture aspects of postmenopausal osteoporosis but do not fully reproduce human fragility fractures, comorbidities, or load-bearing mechanics. Large-animal models and mechanically relevant defect models are needed to evaluate fixation stability, scaffold degradation, load transfer, and long-term performance. Outcome measures should include micro-CT parameters, histomorphometry, vascularization, immune response, mechanical testing, and systemic safety. A third challenge is manufacturing. Complex biomaterial platforms that integrate 3D printing, biologics, nanoparticles, cells, or gene vectors may be difficult to scale reproducibly. Batch-to-batch variation, sterilization, storage, release kinetics, cell or EV potency, and quality control must be defined before clinical translation. For patient-specific scaffolds, the entire workflow from imaging and design to printing, postprocessing, sterilization, and implantation must meet clinical timelines and regulatory requirements. Regulatory pathways are especially challenging for combination products that include a device, drug, and biologic component. Early engagement with regulatory agencies, standardized preclinical testing, and clear mechanism of action claims will be necessary. Relevant regulatory guidance has been issued for genetically modified cell products and combination products (European Medicines Agency, 2020; U.S. Food and Drug Administration, 2022).Claims should also distinguish osteoporosis treatment from osteoporotic defect repair. A biomaterial that improves local bone regeneration may not reduce systemic fracture risk unless tested for that outcome.

Future research should move from material-centered descriptions toward mechanism- and indication-centered design. Promising directions include osteoimmunomodulatory materials, sequential release systems, EV-functionalized scaffolds, AI-guided scaffold optimization, 4D or responsive constructs, and integrated pharmacological-biomaterial regimens. Ultimately, the clinical value of biomaterials in osteoporosis will depend on whether they can improve outcomes that matter to patients: fracture healing, implant stability, pain, mobility, reoperation risk, and quality of life.

## 10 Conclusion

Biomaterial-based strategies offer a promising route to address local repair problems that are not fully solved by systemic osteoporosis drugs. Scaffolds can provide structural support and guide tissue ingrowth; targeted delivery systems can concentrate antiresorptive or anabolic agents at the repair site; biologically derived, cell-based, and EV-functionalized platforms can supply osteogenic, angiogenic, and immunomodulatory signals; and multifunctional systems can integrate several of these functions. The most compelling future approaches will be those that explicitly match material design to osteoporosis-specific pathology rather than simply applying general bone tissue engineering concepts. Nevertheless, most evidence remains preclinical, and translation will require long-term safety testing, standardized osteoporotic models, reproducible manufacturing, and appropriate regulatory strategies. Biomaterials should therefore be considered complementary local interventions that may improve osteoporotic fracture and defect repair when combined with established systemic management.
